# Dengue Fever Outbreak in a Recreation Club, Dhaka, Bangladesh

**DOI:** 10.3201/eid1004.030330

**Published:** 2004-04

**Authors:** Yukiko Wagatsuma, Robert F. Breiman, Anowar Hossain, Mahbubur Rahman

**Affiliations:** * Centre for Health and Population Research, Dhaka, Bangladesh

## Abstract

An outbreak of dengue fever occurred among employees of a recreation club in Bangladesh. Occupational transmission was characterized by a 12% attack rate, no dengue among family contacts, and *Aedes* vectors in club areas. Early recognition of the outbreak likely limited its impact.

Large outbreaks of dengue fever are rarely reported from occupational or institutional settings (*1*), probably because a small proportion of *Aedes* mosquitoes are infected with dengue viruses (*2*), and in dengue-endemic areas, many adults are immune. Dengue has recently reemerged in Bangladesh; in contrast with the situation in countries where dengue has long been endemic, adults appear to become ill with dengue more often than children (*3*).

We investigated an outbreak of dengue fever among employees of a Recreation Club for expatriates in Dhaka. The 636-member club, occupying 92,820 sq ft within a residential area has 107 employees. Initial cases were evaluated by an embassy physician in early October 2001. Club management requested our investigation to define the magnitude of the outbreak and recommend prevention and control strategies.

## Methods

We defined a case of dengue as a febrile illness lasting >3 days during September or October, 2001, with confirmation of dengue infection by presence of antibodies in sera consistent with dengue infection or with presence of dengue viruses in acute-phase sera, detected by reverse transcriptase–polymerase chain reaction (RT-PCR). Cases were identified through occupational absentee logs and through results of initial laboratory testing of acute-phase sera (as ordered by physicians).

Two batches of serum specimens were tested: 18 serum specimens were collected from ill persons by their physicians, and all consenting employees were asked to provide serum specimens 1 month after the outbreak (specimens were collected on November 21, 25, and 26, 2001). Among acute-phase sera, specimens from five patients, collected during the first 5 days of illness, were evaluated for dengue viruses by RT-PCR for serotype-specific dengue viral RNA (*4*).

Acute- and convalescent-phase sera were tested for immunoglobulin (Ig) G and IgM dengue antibodies through capture enzyme-linked immunosorbent assay (*5*,*6*). Specimens with >40 units of IgG or IgM antibodies were considered positive for dengue infection. Ratios of IgM to IgG antibodies of <1.8 were considered indicative of secondary exposure (i.e., previous exposure to dengue virus), and a ratio of >1.8 was considered suggestive of primary (first-time) exposure (*5*).

Written informed consent was taken from each study participant. This study was approved by the ethical review committee of International Centre for Diarrhoeal Disease Research, Bangladesh (ICDDR,B). Interviews with consenting employees were from November 21 through 26, 2002; standardized questionnaires collected sociodemographic information, recent illnesses, febrile illnesses among family members, behaviors and activities, severity of illness, health-seeking behavior, and medications. Detailed information was collected about activities in and around the club. Weight and height were measured; body mass index <20 kg/m^2^ was defined as underweight (*7*). Data were entered into FoxPro, Version 2.6 (Microsoft Corporation, Redmond, WA) and analyzed using SPSS, Version 10.0 (SPSS Inc., Chicago, IL).

A larval survey was conducted on October 20, 2001. All objects containing water (wet containers) were noted, and water from each was sampled and investigated for presence of larvae (larvae were reared to adult stage for species identification).

## Results

One hundred (94%) of 107 employees consented to participate. Dengue fever was confirmed in 13 (12%) of 107 employees, including 12 employees who experienced illness onset within a 10-day period in October (Table 1). One case occurred 10 days earlier (13% attack rate). Twelve (92%) case-employees were male. No severe cases of dengue hemorrhagic fever occurred according to World Health Organization criteria (*8*), but insufficient data were available to rule out grades 1 and 2. One employee was hospitalized; none died. Eleven other employees had febrile illnesses of >3 days duration in September or October (Figure); however, their dengue serologic assays were negative.

**Table 1:** Characteristics of 13 dengue fever patients ^a,b,c^

Case no.	Age	Sex	Mac ELISA (peak outbreak period; Oct 21, 2001)^a^	PCR (Oct 17, 2001; with fever within the past 5 days)	Mac ELISA (Nov 21–26, 2001)	Onset of illness	Occupation
1	26	Male	Primary	-	Primary	01/10/01	Assistant security
2	35	Male	Secondary	-	Secondary	10/10/01	Security guard
3	40	Male	Primary	Negative	Negative	11/10/01	Security guard
4	26	Male	Secondary	-	Secondary	11/10/01	Gardener
5	24	Male	Primary	Negative	Negative	12/10/01	Receptionist
6	22	Male	Secondary	Negative	Secondary	13/10/01	Tennis ball boy
7	27	Male	Negative	Negative	Secondary	14/10/01	Waiter
8	33	Male	Primary	-	Primary	14/10/01	Cook
9	30	Male	Primary	-	Negative	15/10/01	Baker
10	38	Male	-	-	Secondary	15/10/01	Laundry staff
11	38	Female	Secondary	-	Secondary	16/10/01	Laundry staff
12	38	Male	Negative	Den-3	Primary	17/10/01	Confectioner
13	34	Male	-	-	Primary	19/10/01	Gardener

^a^Fever duration > 3 days and serologically confirmed. Those who reported in the table were only for those continuously worked from October 17 to November 2001 (4 employees were no longer working at the club and not available on Nov 21–26, 2001; their Mac ELISA results on October 17 included one specimen positive for primary infection and the others negative). ^b^Serologic survey on Nov 21–26 indicated two more employees, not included in this table, who had dengue antibodies (one primary and one secondary pattern). They did not have symptoms; thus, they were not included as case-employees. ^c^ELISA, enzyme-linked immunosorbent assay; PCR, polymerase chain reaction.

**Figure:**
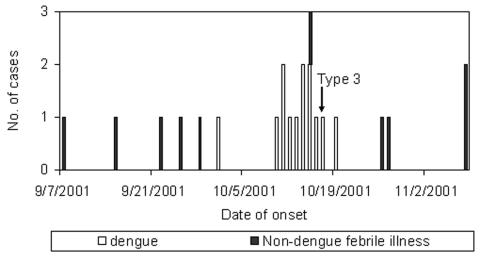
Dates of onset for cases of dengue fever and other febrile illnesses among employees of the expatriate recreation club.

Ten (77%) participants had dengue antibodies in convalescent-phase sera. Samples from three participants had antibodies present in acute-phase sera only. One patient had dengue virus detected by RT-PCR with PCR pattern consistent with dengue serotype 3 (den-3).

Ratios of IgM to IgG suggested first-time infection among seven (54%) participants and secondary infection in six participants. In addition to the 13 cases of dengue, samples from two employees had dengue antibodies detected during the November serosurvey; the employees had not been ill. One had evidence of primary infection and one of secondary infection on the basis of the IgM/IgG ratio.

In addition to fever in all 13 dengue-positive case-employees (as required by the case definition), 11 (85%) had headache, 5 (39%) had myalgias, and 3 (23%) reported gum bleeding. Nine (69%) patients sought care from clinic or hospital; four persons with dengue did not seek care and reported using medication to alleviate symptoms.

Among 308 family members residing with the 100 employees, 21 (7%) family members were reported to be ill with fever during September and October. The rate (14%) of febrile illnesses among family members was significantly higher among 11 employees with febrile illness with negative dengue assays than among the 13 dengue patients (2%; p = 0.04; Table 2).

**Table 2:** Illness reported among employees and family members during the outbreak period, September–October 2001

Employee categories	Family members with history of febrile illnesses during outbreak period	Dengue laboratory test confirmation done for febrile illnesses among family members
13 employees with confirmed dengue	2.0% (1/51)^a^	No laboratory confirmation done
76 employees who did not have dengue and did not have a febrile illness during outbreak period	6.5% (14/215)^a^	6 febrile patients had laboratory tests and 2 were serologically confirmed as dengue fever at commercial pathology laboratories
11 employees who did not have dengue and who had a febrile illness during outbreak period	14.3% (6/42)^b^	No laboratory confirmation done
Total employees = 100	6.8% (21/308)^a^	

^a^Number of febrile illnesses/number of family members. ^b^p = 0.04 when compared with the percentage of febrile illness among family members of case-patients.

The first three dengue cases occurred among security guards. They spent most working hours on the perimeter of the club, particularly around the east and west sides. Overall 3 (23%) of 13 security guards were cases compared with 10 (11%) of 87 other participating employees. Three case-patients (gardener, receptionist, and tennis ball boy) shared significant time, i.e., > 1 hour, with the first three case-patients around the main entrance, as well as within the canteen, west side and laundry room, and changing room areas. Subsequent case-patients represented a wide variety of occupations, and these employees spent time in a variety of locations around the club. The staff did not have living quarters at the club.

Epidemic dengue did not occur among club members. While surveillance was not systematically conducted, 2 (0.3%) of 636 members were known to have had dengue fever during October.

We compared data from case-employees with 76 other employees (noncase-employees) who did not have febrile illnesses during September or October. Age distribution was similar for case-employees (mean 32 years) and noncase-employees (mean 36 years). Differences in sex, duration of employment, body mass index, working hours, time spent indoors or outdoors, medications, or smoking were not significant.

Case-employees (100%) were more likely than noncase-employees (83%) to spend any time within the canteen area during break time (Kendall’s τ p < 0.01). Case-employees (62%) were also more likely than noncase-employees (33%) to come to the club by walking (odds ratio = 3.3; 95% confidence interval 1.0 to 11.0; p < 0.05). Mosquito repellent use was associated with a slightly reduced likelihood of dengue infection (0% in case-employees and 9% in noncase-employees; Kendall’s τ, p < 0.05).

A total of 23 larvae-positive containers were found among 34 wet containers (container index = 68) (Table 3); 364 larvae (103 *Ae. aegypti* and 261 *Ae. albopictus*) were identified in stagnant water covering surface lids of 20 metal drums used for security (to block traffic) at the west perimeter.

**Table 3:** Survey of wet containers for *Aedes* larvae within and outside of the club

Place	Container type	No. of wet containers	No. of positive containers	No. of larvae	Mosquito species		
					*Aedes aegypti*	*Ae. albopictus*	Others
Outside of premise boundary	Metallic drum cover	26	20	364	103	261	0
Outside of club building	Plastic glass	1	1	19	0	19	0
	Manhole cover	6	1	4	4	0	0
Inside of club building	No wet container found	0	0	0	0	0	0
Rooftop of club building	Stagnant water on rooftop floor	1	1	5	5	0	0
Total		34	23^a^	392	112	280	0

^a^ Overall container index (CI) was 68 (23/34 x 100).

## Discussion

Intense focal transmission of dengue viruses occurred within an occupational setting in a community experiencing endemic dengue. Focal intensity is highlighted by a 12% attack rate among employees for a 2-week period, compared with no known cases of dengue among nuclear family members. In other Asian countries where dengue is established and where multiple serotypes circulate, outbreaks of dengue fever in occupational settings are uncommon since adults are usually immune; disease is highest in children (*8*). Other febrile illnesses were occurring simultaneously among employees; these illnesses were probably caused by another communicable disease, as suggested by a higher attack rate of febrile illnesses among family members of febrile employees who did not have dengue.

Dengue viruses are transmitted by infected mosquitoes during feeding, which may occur several times a day, for a 1- to 4-week lifetime (*9*). The larval survey showed that *Aedes* mosquitoes were present in working areas. High concentrations of *Aedes* larvae in water on security drum surfaces may have provided a mechanism for this outbreak. The first three cases were among security guards who were often in close proximity with the security drums. The guards also spent time inside the club (eating, praying, changing clothes, and taking breaks). The security guards may have been exposed to dengue while on patrol outside of the club, and once infected, transmitted dengue viruses to adult *Aedes* mosquitoes feeding on them while they spent time inside the club. Mosquitoes, thus infected, were able to quickly infect other employees working or resting within the club, perhaps within the staff canteen, resulting in a burst of illnesses. The end of intense transmission coincided with recognition of the outbreak, aggressive use of insecticides, and removal of breeding sites.

The outbreak likely resulted from conditions which promoted rapid transmission of dengue viruses, such as high vector density and many susceptible (nonimmune) people within close quarters. Primary infection among seven cases supports the notion that dengue has recently emerged in Bangladesh. No club members were case-patients, reflecting the importance of duration of exposure in risk for transmission.

Early recognition of the outbreak may have helped limit its impact (*10*). Institutional or systematic monitoring of suspected cases, i.e., surveillance, supported by prompt laboratory confirmation, may help to contain such outbreaks. Integrating and targeting vector control as soon as a cluster of cases is detected can suppress transmission and minimize numbers of cases.
